# Eye movements along the establishment of functional stimuli classes

**DOI:** 10.1002/jeab.70016

**Published:** 2025-06-30

**Authors:** Nicolau K. Pergher, Edson M. Huziwara, Gerson Y. Tomanari

**Affiliations:** ^1^ Universidade de São Paulo Brazil; ^2^ Universidade Federal de Minas Gerais Brazil; ^3^ Instituto Nacional de Ciência e Tecnologia sobre Comportamento, Cognição e Ensino (INCT‐ECCE) Brazil

**Keywords:** functional classes, observing responses, visual discrimination

## Abstract

The present study analyzed the eye movement patterns of five typically developed adults who were exposed to a series of simple discrimination training tasks with reversals in the contingencies of reinforcement that led to the formation of functional stimulus classes. Two studies were planned. In Study 1, two visual stimuli were used to carry out one training phase and three consecutive reversals. In Study 2, the phases were repeated but four‐stimuli functional classes were established. In the second study, the selective observing responses to stimuli of functional classes following the reversal of the first stimulus were analyzed. The results showed shifts in the duration of observing responses as the discriminative functions of the stimuli were established and reversed. Unlike the existing literature, our study reveals that some participants maintain longer observing responses to S– than to S+. Moreover, following the reversal of the first stimulus, observing responses to all other stimuli of the same functional class change immediately and accordingly. These findings deepen our understanding of discriminative stimulus control and shed light on the role of observing responses to stimuli composing functional classes.

In the last 20 years, a growing number of experiments in behavior‐analytic literature have described eye movement patterns in different learning tasks (e.g., Braaten & Arntzen, 2022; Dube et al., 2010; Huziwara et al., 2016; Perez et al., 2015; Pessôa et al., 2009; Sadeghi & Arntzen, 2018; Steingrimsdottir & Arntzen, 2016). For instance, Dube et al. (2006) described eye movements in a matching‐to‐sample (MTS) procedure using four‐component sample stimuli presented in a 2 × 2 array. This stimulus disappeared when the sample display area was clicked, and three comparison stimuli were presented after a 1‐s delay. Importantly, one of the comparisons was identical to one of the components of the sample. In this task, participants were reinforced for choosing the comparison stimulus that was previously presented as a sample component. The results showed that participants who spent more time observing all components of the sample stimuli achieved a higher percentage of correct responses.

Hansen and Arntzen (2021) analyzed eye movements when different MTS training structures (i.e., one‐to‐many, many‐to‐one, and linear series) were used to establish stimulus equivalence classes. To this end, participants formed three groups and were exposed to one of the three MTS training structures to establish six 3‐member classes. In general, successful results on equivalence class formation were accompanied by longer observing durations of the sample stimuli and shorter observing durations of the comparison stimuli. In addition, longer observing durations of the correct comparison than of the incorrect one in each trial were described for all participants regardless of the results for equivalence class formation.

Certainly, the stimulus control over eye movements has provided useful measures for a more fine‐grained examination of the learning process in several behavioral tasks (e.g., Dube et al., 2006, 2010; Huziwara et al., 2015, 2016; Pessôa et al., 2009; Schroeder, 1969, 1970, 1997; Schroeder & Holland, 1968, 1969; Tomanari et al., 2007). Moreover, because directing participants' attention to the relevant aspects of the training task is a fundamental part of the discrimination acquisition process (Serna & Carlin, 2001), eye movement analysis could be crucial for proposing more efficient teaching procedures.

Most published experiments analyzed eye movements in conditional discrimination tasks (e.g., Braaten & Arntzen, 2022; Dube et al., 2006, 2010; Hansen & Arntzen, 2021; Huziwara et al., 2016; Perez et al., 2015; Sadeghi & Arntzen, 2018; Steingrimsdottir & Arntzen, 2016), whereas less attention has been given to simple discrimination training tasks (e.g., Huziwara et al., 2015; Pessôa et al., 2009; Tomanari et al., 2007). For example, Huziwara et al. (2015) evaluated eye movement patterns in simultaneous and successive simple discrimination training. Using 12 paintings by Hiroshige (1986) as visual stimuli, three participants were given simultaneous discrimination training followed by successive discrimination training. Another three participants were exposed to this training procedure in the opposite order. In the simultaneous discrimination training, a trial began by presenting two stimuli (i.e., S+ and S–) in two of the screen's four corners. The correct response was choosing the S+ stimulus by pressing a key corresponding to the location where the S+ was presented. No time limit was imposed to complete the trial (i.e., the stimuli remained available until the participant emitted the choice response).

In the successive discrimination training, a single stimulus was presented in one of four positions on the screen for up to 5 s. Whenever the S+ was presented, the correct response was pressing the key corresponding to the location where the stimulus was presented within the 5‐s time limit, whereas not pressing the key within the stipulated time was considered incorrect. Whenever the S– was presented, the correct response was not pressing the key corresponding to the location where the S– was presented within the 5‐s time limit, and an incorrect response was pressing the key within the stipulated time. The results showed that the stimulus observing durations in successive discrimination training were higher than in simultaneous discrimination training regardless of the training sequence. In addition, on average, the observing durations were higher for S+ than for S– in the simultaneous discrimination training. Conversely, it was higher for S– than for S+ in the successive discrimination training.

Tomanari et al. (2007) obtained similar results by analyzing the eye movement patterns of five participants who were exposed to successive simple discrimination training under two conditions. In the first condition, the S+ and S– stimuli were presented successively in components lasting 10 s under a multiple schedule. Pressing the space bar on the computer keyboard was reinforced on a variable interval (VI) 5‐s schedule whenever S+ was present and did not produce any programmed effect (i.e., extinction) whenever S– was present. The same reinforcement and extinction schedules were in place in the second condition but under a mixed schedule. More specifically, the discriminative stimuli remained covered by a gray square. Removing the square to observe the stimulus (S+ or S–) was enabled by pressing a button on the computer screen (i.e., a manual observation response). In the multiple schedule, participants observed S+ longer than they observed S–. Conversely, in the mixed schedule, manual observation responses (i.e., pressing the button) occurred longer in S– than in S+ components.

Tomanari et al. (2007) attributed the longer observing duration of the S+ in the multiple schedule to its discriminative function. Their study used a black lowercase Greek letter lambda as S+, whereas a black uppercase Greek letter Xi was used as S– across all sessions for all participants. Then, the visual properties of the stimuli that were unrelated to their discriminative function might also explain the observing pattern they described. A necessary control to disentangle the effects of discriminative function from the visual properties of the stimuli on observing duration would be using lambda as S+ for half of the participants and as S– for the other half. Alternatively, implementing reversals in reinforcement contingencies, as conducted by Vaughan (1988), could provide further experimental control to investigate this issue. This approach would allow an evaluation of whether selective observing responses to each stimulus change when their discriminative functions are reversed.

Simple discrimination with reversals in the reinforcement contingencies is used to evaluate the acquisition of complex behavior patterns, such as abstraction (e.g., Holth, 2017; Watanabe et al., 1995) and functional class formation (e.g., Canovas et al., 2015, 2019; Goldiamond, 1966; Lionello‐DeNolf et al., 2008; Postalli et al., 2015). For example, Lionello‐DeNolf et al. (2008, Experiment 1) conducted an experiment using simultaneous simple discrimination training with reversals to establish functional classes in typically developing preschool children. Three stimulus pairs (i.e., A1, A2; B1, B2; and C1, C2) were divided into two sets of three stimuli each (i.e., Class 1 and Class 2). Generally, each trial presented two stimuli from the same pair (e.g., A1 and A2). Participants were asked to choose the S+ in each trial. In the first training phase, stimuli from Class 1 served the S+ function and stimuli from Class 2 served the S– function. When the stipulated learning criterion was achieved, the stimuli discriminative functions were reversed. That is, stimuli from Class 1 became the S– and stimuli from Class 2 became the S+. In this context, A1, B1, and C1 and A2, B2, and C2, shared the same discriminative functions (i.e., S+ or S–) throughout the sessions.

If this training gave rise to two classes of functionally equivalent stimuli, then reversing the discriminative function for one (e.g., A1) would lead to the function reversal for the remaining stimuli in the same set (e.g., B1 and C1) without the need for direct training (Vaughan, 1988). To evaluate this hypothesis, Lionello‐DeNolf et al. (2008, Experiment 1) presented only two stimulus pairs (e.g., A1, A2; B1, B2) in the first few sessions following the occurrence of a reversal. After that, C1 and C2 were reintroduced. The functional class formation would be demonstrated if the responses to this third stimulus pair were consistent with the reversed contingencies even before those responses produced consequences. That is exactly what has happened: All six participants showed results that suggested functional class formation in Lionello‐DeNolf et al.'s study as well as in others that have followed (e.g., Canovas et al., 2019; McIlvane et al., 1990; Postalli et al., 2015).

As previous studies have demonstrated, selective observing responses occur when visual discrimination is established, typically with longer eye fixations on S+ than on S− (e.g., Hansen & Arntzen, 2021; Huziwara et al., 2015; Tomanari et al., 2007). Additionally, research has shown that functional stimulus classes emerge through reversals of simple discriminations (e.g., Canovas et al., 2019; Lionello‐DeNolf et al., 2008; McIlvane et al., 1990; Postalli et al., 2015; Vaughan, 1988). However, how selective observing evolves throughout the discrimination reversals that give rise to functional stimulus classes remains unknown.

The present study seeks to fill this gap by investigating how exposure to simple discrimination training with reversals in reinforcement contingencies modulates eye movement patterns in human participants. Two studies were planned. In Study 1, three participants performed a simple discrimination task in which two visual stimuli were used in one training phase and three consecutive reversals. More specifically, A1 was related to a variable ratio [VR] 10 reinforcement schedule, and A2 was related to a VR 10 punishment schedule in the training phase. Contingencies were reversed after participants achieved the stipulated learning criterion. In Study 2, two other participants performed a task in which eye movements were analyzed, and two four‐stimuli classes originated from a sequence of discrimination reversals.

## METHOD

### Participants

Five adults, aged between 20 and 38 years (male = 2; *M* = 27.6 years, *SD* = 7.17), with no previous experience in behavior‐analytic experimental research and without noticeable visual deficits, participated in the experiment. Additional demographic information, such as race and ethnicity, was not collected. Three participants were given the experimental task designed for Study 1, and the remaining two were given the experimental task designed for Study 2 (for the task description, see the Procedure section). Before starting the task, participants read and signed a consent form that presented information about the general aspects of the task and its estimated duration.

### Setting and apparatus

The data were collected in a well‐lit 3‐ × 4‐m room with a low noise level. To collect observing response data, the computer software described in Tomanari et al. (2007) running on an Apple Macintosh 6160 computer with a 15‐in. (~38 cm) screen was used to show stimuli, record responses, and deliver reinforcement to the participants.

During the experimental task, an ISCAN model RK‐426PC tracking system was used to register eye movements. The data produced by this system corresponded to a video of the participant's visual field (20 × 20) in which an overlapping cursor indicated the location of sight fixation with 0.3° precision. The visual stimuli dimensions were approximately 2 × 2 cm, and the screen was approximately 65 cm from the participant's eyes. In this case, the maximum size for the visual stimuli in terms of visual angle was 2 × 2.

The Video Frame Coder software transformed the video into a continuous sequence of frames. These frames were coded to indicate the direction of the participant's gaze moment by moment in the session to obtain the time spent looking at each stimulus (i.e., observing duration). A second independent observer coded the frames of 20% of all sessions. Observer agreement—the number of agreements divided by the sum of agreements and disagreements and multiplied by 100—was greater than 98%.

### Stimuli

Table 1 presents the eight Greek letters that were used throughout the experiment. These stimuli were presented in the upper‐left corner of the screen, 1 cm from the edge, successively according to the experimental schedule described in the Procedure.

**TABLE 1:** Visual stimuli and alphanumeric identification (ID) used in the experiment.

Class 1		Class 2	
Alphanumeric ID	Greek letter	Alphanumeric ID	Greek letter
A1	λ	A2	Ξ
B1	ξ	B2	Θ
C1	φ	C2	Φ
D1	ω	D2	δ

### Procedure

#### Calibration

Sessions began with an apparatus calibration routine lasting approximately 10 min. In this routine, the participant's pupil illumination and corneal reflection were adjusted using ISCAN Raw Movement Data Acquisition software commands. The participants were asked to keep their head still and to fix their visual gaze on some cross‐shaped signs in the monitor's corners. The calibration routine ended when the overlapping cursor generated by the ISCAN accurately represented the participant's visual gaze. After calibration, the head movement restriction was discontinued. However, if the participant moved their head abruptly or if pupil illumination and corneal reflection changed, the calibration could be disrupted during the session. These disruptions were easily identifiable by erratic cursor movements or the absence of the cursor on the screen. The calibration routine was repeated whenever necessary.

#### Pretraining

Before the beginning of the experimental task, the following text (written in Portuguese) was shown to the participants: “Please read the instructions aloud. First, we will test the computer's keyboard. Then, after pressing ‘Continue’, please follow the instructions below.”

When the participants pressed the “Continue” button, a visual stimulus was presented in the upper‐left corner of the screen. In the upper‐right corner, a point counter was presented. This counter was empty during the pretraining phase. However, it showed the total points accumulated by the participant during each session of the actual experimental task. Finally, a rectangle (10 × 4 cm) in the lower‐right panel of the screen was used to present the instructions for the task.

During the pretraining phase, participants were shown alternating figures of a red ball and an empty glass in the upper‐left corner for 60 s. More specifically, these figures alternated every 5 s. In addition, they were asked to perform space bar press responses while the pictures were presented. If the participants pressed the space bar at least five times within the 60 s, the message “The program and the equipment are OK. Press ‘Continue’ when you are ready to start the task.” was presented to them. When the participants pressed the “Continue” button, the actual experimental task was begun.

This pretraining was chosen to establish the space bar as the manipulandum in the task. Additionally, participants would be expected to infer that changes in the visual stimuli were unrelated to the space bar responses. Because of its purpose, no specific responses were reinforced during the pretraining phase.

#### Study 1: Multiple reinforcement schedule using two visual stimuli

The purpose of this study was to establish two Greek letters displayed in the upper‐left corner of the screen as discriminative stimuli for pressing the space bar to earn points. During the task, the following instructions, written in Portuguese, were presented: “Pressing the space bar will sometimes earn points. After the session, you will be paid 5 cents for every 10 points.”

Sessions comprised 12 A1 components and 12 A2 components presented in a semirandom sequence; neither could be presented more than three times consecutively. Components lasted 10 s; thus, A1 or A2 stimuli were not presented for more than 30 s in a row. In addition, sessions were carried out three or four times daily, depending on the participants' availability.

During the training phase, a variable‐ratio (VR) 10 schedule was in effect when the A1 stimulus, a black lower‐case Greek letter lambda, was presented (i.e., S+ component). Thus, for every 10 responses, on average, 10 points were added to the point counter. In addition, a beep sound was presented and a small white square below the point counter turned red for 0.5 s when points were earned. It is important to note that by emitting a high response rate, participants' responses could produce several occurrences of reinforcement within a single 10‐s component. However, the response counter was restarted at each new component. In other words, responses in the first A1 component that did not produce the consequences were not considered for the response counting in the second A1 component, even when the components were contiguous.

When the A2 stimulus (a black upper‐case Greek letter Xi) was presented, a punishing schedule was in effect (i.e., S− components). In this case, performing 10 responses on average was followed by a withdrawal of 10 points. Moreover, a buzzing sound was presented and the small white square turned black for 0.5 s when points were lost. As in A1 components, participants could produce several occurrences of losing points within a single component by emitting a high response rate. Notably, due to the possibility of losing points throughout the session, participants began the session with 500 points.

Finally, there were no specific consequences for participants who did not respond during the task. In these cases, stimuli were alternated in the upper‐left corner without reinforcement or punishment, depending on the component in effect during the absence of responses.

The frequencies of space bar responses across different components were recorded. The discrimination index (DI) was used to determine relative stability in the data due to the high values and marked variability in the number of responses across sessions. The DI was calculated at the end of each experimental session (12 A1 components and 12 A2 components). This index was calculated by dividing the number of responses when S+ was displayed by the total number of responses during both S+ and S–. This training lasted until participants presented a DI equal to or greater than 0.70 for three consecutive sessions, as in Tomanari et al. (2007). Then, the reinforcement contingencies were reversed; that is, responses to A2 stimuli were reinforced and responses to A1 stimuli were punished. One training phase and three consecutive reversals with stimuli A1 and A2 were included in Study 1. Participants who did not meet the DI criterion in the training phase within 20 sessions were dismissed.

The use of both reinforcement and punishment contingencies in this experiment requires justification. A previous study (Pergher, 2007, Experiment 1) using reinforcement and extinction schedules failed to establish Greek letters as discriminative stimuli for space bar pressing. In that study, participants exhibited high response frequencies to all stimuli, suggesting that the minimal effort required to press the bar might have led to indiscriminate responding throughout the session, rendering stimulus presentation irrelevant. Consequently, punishment components were introduced in the present study to generate a discriminated response pattern.

#### Study 2: Multiple reinforcement schedule using two visual stimulus sets

Prior studies (e.g., Canovas et al., 2019; Lionello‐DeNolf et al., 2008; McIlvane et al., 1990; Postalli et al., 2015) have reported successful outcomes in discrimination learning when human participants were exposed to experimental procedures using sets of stimuli as S+ and S–, alongside reinforcement and extinction schedules—that is, without the need to employ punishment schedules. These successful results were achieved even in experimental procedures that required responses with minimal effort, such as mouse clicks.

Based on those preparations, two 4‐stimulus sets (i.e., A1, B1, C1, and D1; A2, B2, C2, and D2) were used to establish a discriminated response pattern in Study 2. All stimuli were presented in a semirandom sequence with up to three consecutive presentations of the same set of stimuli. Each session comprised 12 Class 1 and 12 Class 2 components, so each stimulus was presented three times in each session. Components lasted 10 s. Similar to Study 1, participants were asked to achieve a DI equal to or greater than 0.70 for three consecutive sessions to produce the reversal of contingencies. One training phase and three consecutive reversals were carried out.

In Study 2, a VR 10 was in effect in S+ components and extinction was in effect in S– components; that is, P4 did not lose points for pressing the space bar in the S– components. Due to the results obtained by P4, reinforcement and punishment schedules were reintroduced for P5. Then, for Participant P5, a VR 10 reinforcement schedule and a VR 10 punishing schedule were in effect in the S+ and S– components, respectively.

## RESULTS

**FIGURE 1:**
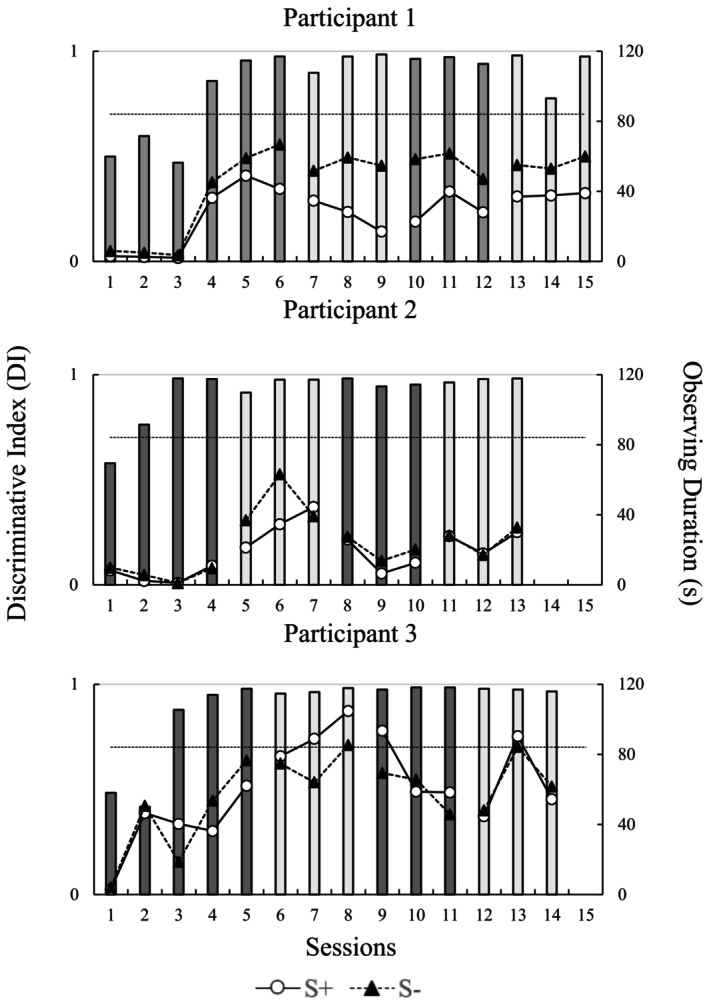
Discriminative index (left ordinate axis) and observing durations (right ordinate axis) for S+ and S– stimuli throughout the sessions for participants in Study 1. Circles and triangles represent durations of the observing response to S+ and S– stimuli, respectively. Dark gray bars represent DI in sessions where A1 served the S+ function. White bars represent DI in sessions where A2 served the S+ function. The dashed horizontal line represents the DI criterion to trigger a reversal of the reinforcement contingencies (DI = 0.70).

Figure 1 presents the DI and the observing duration per session for participants in Study 1. The bars, which ranged from 0 to 1, represent the DI. In addition, dark gray bars represent DI in sessions where A1 served the S+ function. White bars represent DI in sessions where A2 served the S+ function. The dashed horizontal line represents the DI criterion for reversing reinforcement contingencies. Circles and triangles represent durations of observing response to S+ and S– stimuli, respectively. The figure's upper, middle, and lower panels show the performances of P1, P2, and P3, respectively.

P1 achieved DIs close to 0.50 in the first three sessions. In the fourth session, a 0.86 DI was achieved, which means that 86% of the responses (i.e., pressing the space bar) occurred in the presence of the S+ stimulus. In all the following sessions, the DI values were above 0.70. Considering the programmed learning criterion, reversals in the reinforcement contingencies were performed every three sessions following the fourth session (see dark gray and white bars). Decreases in the DI were observed only in Sessions 7 and 14.

Related to the observing durations, it is noteworthy that P1 observed S– for a longer duration than S+, regardless of the stimulus (i.e., A1 or A2) that served this discriminative function throughout the sessions (i.e., triangles above the circles in all sessions). For example, whenever Stimulus A1 served as S+ (i.e., dark gray bars), P1's observing durations were higher for A2 than A1. Conversely, whenever Stimulus A2 served as S+ (i.e., white bars), the observing durations were higher for A1 than for A2. It is also important to note that the observing durations of both stimuli increase with the increase in DI. In the first three sessions, the total observation time of the stimuli is close to 0 s. Each stimulus was usually observed for 40 or 50 s from the fourth session to the end of the sessions.

Participant P2 needed only 13 sessions to complete the experimental procedure. Discriminative indexes above 0.70 were obtained at the second session and remained close to 1.0 throughout the procedure. Considering the learning criterion, reversals in the reinforcement contingencies were performed in the 5th, 8th, and 11th sessions (see dark gray and white bars).

The observing duration data showed that, in most sessions, participant P2 spent similar amounts of time observing each stimulus. This observation pattern changed only in Sessions 5 and 6 when the first reversal in reinforcement contingencies occurred. In those sessions, Stimulus A2 served as S+ (i.e., white bars) and the observing durations were higher for A1 (S–) than for A2. Furthermore, in the first reversal, it was also possible to observe an increase in the time spent observing the stimuli. In the first four sessions, the time spent observing each stimulus varied between 5 and 10 s, but in the first reversal, it varied between 15 and 65 s. In the second and third reversals, P2 observed each stimulus for approximately 15 s in each session.

Similarly to P2, P3 achieved a DI close to 0.90 in the third session and remained close to 1.0 throughout the procedure. Eye movement data showed that both stimuli were observed with similar durations in the later sessions; however, the A2 stimulus was observed for longer periods than A1 from the middle of the initial training to the end of the first reversal. More specifically, the triangles are above the circles in Sessions 4 and 5 when A2 was the S–; in Sessions 7 and 8, circles are above the triangles when A2 was the S+. It is important to highlight that P3 was the participant who spent the longest time observing the stimuli presented in the task. For instance, in the eighth session, stimuli S– and S+ were observed for 80 and 115 s, respectively.

**FIGURE 2:**
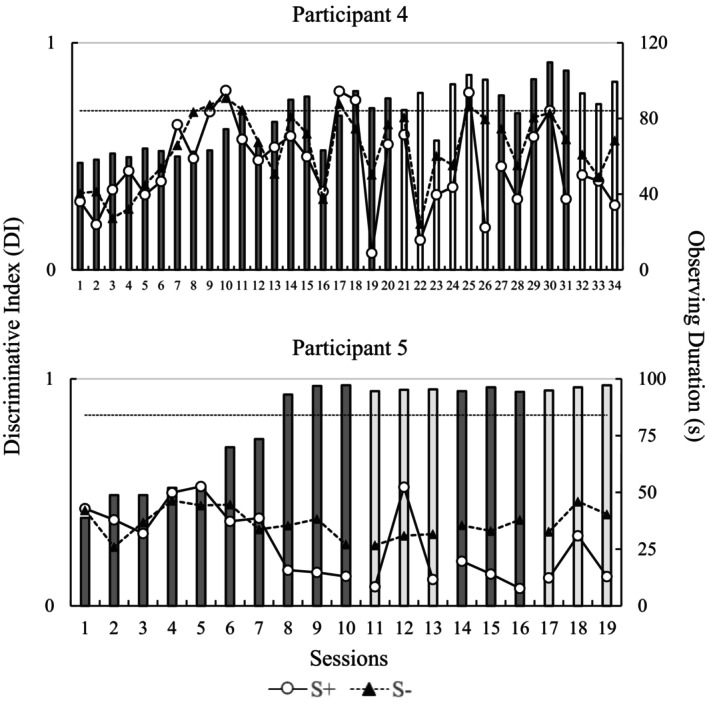
Discriminative index (left ordinate axis) and observing durations (right ordinate axis) for S+ and S– stimuli throughout the sessions for participants in Study 2. Circles and triangles represent durations of the observing response to S+ and S– stimuli, respectively. Dark gray bars represent DI in sessions where Class 1 stimuli served the S+ function. White bars represent DI in sessions where Class 2 stimuli served the S+ function. The dashed horizontal line represents the DI criterion to trigger a reversal of the reinforcement contingencies (DI = 0.70).

The discriminative index and the observing duration per session for participants in Study 2 are presented in Figure 2. Once again, dark gray bars represent DI in sessions where A1 exerted the S+ function. White bars represent DI in sessions where A2 exerted the S+ function. The dashed horizontal line represents the DI criterion for reversing reinforcement contingencies. Empty circles and filled triangles, respectively, represent the duration(s) of S+ and S– observing responses. The figure's upper and lower panels show the performances of P4 and P5, respectively.

Participant P4 needed 34 sessions to complete the experimental procedure. Until Session 9, pressing the space bar occurred during all the sessions regardless of the stimulus class presented. After Session 10, responses were systematically emitted at a higher frequency in the presence of the S+ stimuli. Some disruptions in the DI (e.g., Sessions 12 and 16) delayed implementing the first reversal. Therefore, the DI‐based learning criterion for the initial training phase was achieved after 20 sessions for P4. The criteria for the subsequent reversals were achieved after six, five, and three sessions, respectively.

Related to the observing duration data, stimuli from both classes were observed for similar durations in most sessions. However, there are some sessions where a pronounced difference was observed. For instance, S– stimuli were observed for longer than S+ stimuli in Sessions 19 and 31 when the S– stimuli were Class 2 stimuli. The same observing duration pattern was seen in Sessions 26 and 34 when the S– stimuli were Class 1 stimuli. In all cases, the more observed stimuli class was the S– class. It is also important to note that the observing durations of S– and S+ stimuli increase from Session 3 to Session 10.

For P5, pressing the space bar regardless of the presented stimulus class occurred until Session 5, resulting in a DI close to 0.50. A discriminative index above 0.70 was achieved in Session 8 for the first time. Afterward, DI was close to 0.90 in all remaining sessions. The eye movement data showed that P5 observed the S– longer regardless of the class serving this discriminative function. For example, observing durations were higher for S– than for S+ from Sessions 14 to 16 when the S– stimuli were Class 2 stimuli. The same observing duration pattern was seen from Sessions 17 to 19 when the S– stimuli were Class 1 stimuli. Interestingly, the observing durations of both stimuli decreased with the increase in P5's DI, which is the opposite of the observing pattern described for P1 (see data from the last five sessions of the initial training).

**FIGURE 3:**
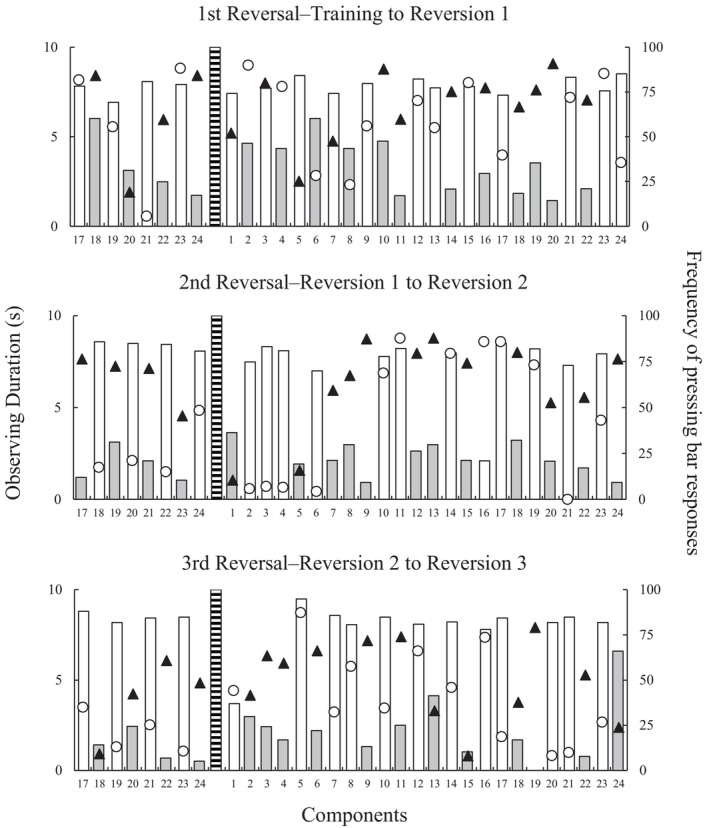
Response frequency and observing duration for P4 in the last eight 10‐s components of the session that preceded reversals and the 24 components of the first session after each of the three reversals. Circles and triangles represent durations of the observing response to S+ and S– stimuli, respectively. Additionally, white and gray bars represent the frequency of space bar responses in S+ and S– components, respectively. Dashed bars indicate the division between sessions.

Data obtained in the last eight components of the sessions that preceded reversals and the 24 components of the first sessions after reversals for P4 are presented in Figure 3. The upper, middle, and lower panels show data for the first, second, and third reversals. Circles and triangles represent durations of observing response to S+ and S– stimuli, respectively. White and gray bars, respectively, represent the frequency of space bar responses in S+ and S– components. Finally, dashed bars indicate the division between sessions.

The frequency of responses on the S– components indicates the functional class formation from the second reversal. Specifically, in the first reversal, responses to the S– decreased only after the fifth component presentation (see gray bars in the upper panel). On the other hand, in the second and third reversals, this decrease occurred in the first or second component (see gray bars in the middle and lower panels). That is, the participants only needed to be exposed to some of the components of each class to adapt their response pattern to the new contingency. The number of responses to S+ was high in most components. The only exception occurred in the third reversal, first component (see the white bar in the lower panel). This result was expected, considering that responses to stimuli of this class were punished before reversal occurred.

Regarding the eye movements, the data do not indicate systematic differences in the observing duration of the S+ and S– components in most cases. The only exception occurred in the final components of the second reversal (see circles and triangles in the middle panel). In this period, it is possible to verify that the observing duration is longer for S– than for S+.

**FIGURE 4:**
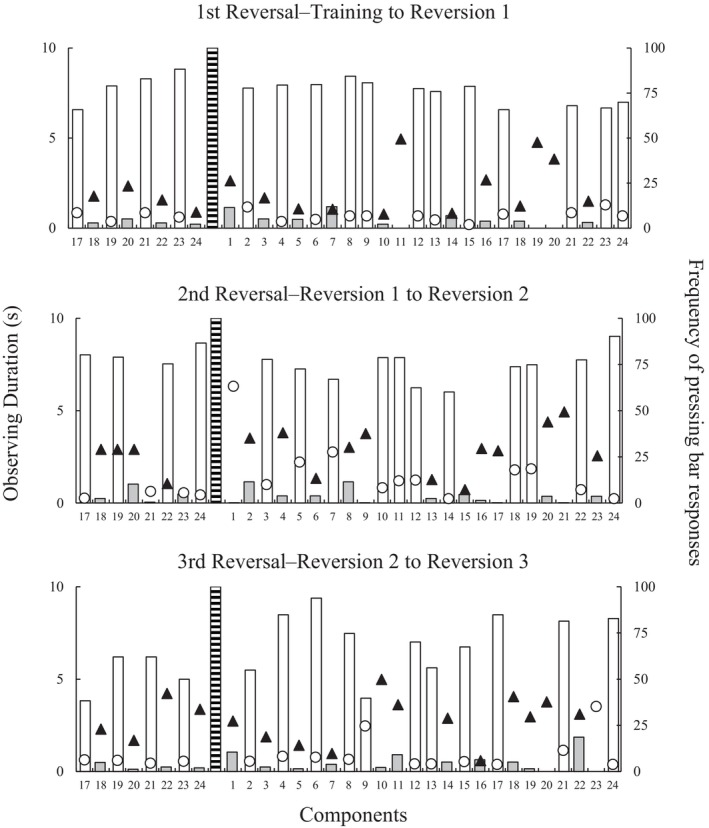
Response frequency and observing duration for P5 in the last eight 10‐s components of the session that preceded reversals and the 24 components of the first session after each of the three reversals Circles and triangles represent durations of the observing response to S+ and S– stimuli, respectively. Additionally, white and gray bars represent the frequency of space bar responses in S+ and S– components, respectively. Dashed bars indicate the division between sessions.

The same analysis of response frequency and observing duration in pre‐ and postreversal sessions was presented for P5 in Figure 4. Considering that the decrease in responses occurred starting with the first S– component after the reversals, it seems possible to argue that the functional class formation arose from the first reversal for P5. In turn, the number of responses to S+ was high in most components in the first and second reversals. The only exception occurred in the first component of the second reversal when only one response occurred. Once again, it was an expected result considering that responses to stimuli of this class were punished before reversal occurred. Regarding the number of responses to S+ in the third reversal, however, it is possible to verify a decrease at the end of the pre‐reversal session (see the white bar in the lower panel) and a considerable variation in the first postreversal session, including a component in which no responses occurred.

Regarding the eye movements, the observing durations presented by P5 are shorter than those presented by P4. In addition, it is possible to argue that the S– was observed for longer periods than S+ in the final components of pre‐reversal sessions and the first components of postreversal sessions.

## DISCUSSION

The present experiment sought to replicate previous findings on functional class formation (Vaughan, 1988) in humans (e.g., Canovas et al., 2019; Lionello‐DeNolf et al., 2008; McIlvane et al., 1990; Postalli et al., 2015) and extend the literature by examining changes in observing responses during simple discrimination training and reversals as functional classes developed. Although prior research has established the role of discriminative stimulus reversals in functional class formation, less is known about how observing responses—especially when influenced by contingency reversals—contribute to the development of these classes in human participants.

Particularly, we sought to (1) replicate functional class formation in human participants and (2) examine changes in observing responses during simple discrimination training and reversals as functional classes developed. In Study 1, two visual stimuli (A1 and A2) served as S+ and S–, and Study 2 used two sets of four S+ and four S–. Most participants in both studies showed clear changes in stimulus observing duration as discriminative functions were established. For P1, this increase coincided with establishing the discriminative function of the stimuli in the training phase (see increasing DI values in Figure 1). For P2 and P3, a similar change in observing response time occurred when the discriminative functions of the stimuli were reversed for the first time in the procedure (i.e., First Reversal). Finally, for P4, observing response time increased in the initial sessions of the procedure; however, it did not coincide with the increase in DI.

Research with nonhuman subjects suggests a correlation between establishing a stimulus's discriminative function and observing duration (e.g., Dinsmoor, 1985; Dinsmoor et al., 1982; Wyckoff, 1952, 1969). This relation may account for the simultaneous changes in observing patterns during stimulus control establishment and reversals in our experiment. However, given the diverse procedures and varied results across studies, a more detailed investigation of the variables influencing observing responses in human participants is needed. Such research could clarify the mechanisms linking stimulus observation and discriminative control in humans.

Despite using continuously available discriminative stimuli, similar to procedures for a multiple schedule, our experiment produced different results from those of Tomanari et al. (2007), where S+ observation was longer than S–. In our study, most participants consistently observed S– longer, which suggests that contingency reversals may alter observing behavior in a way that cannot be attributed solely to the visual aspects of stimuli. This shift further emphasizes the need for more controlled procedures when examining observing responses, particularly concerning the specific contingencies employed. Notably, whereas Tomanari et al. used reinforcement and extinction schedules, we incorporated reinforcement and punishment schedules for some participants (P1, P2, P3 in Study 1; P5 in Study 2). This procedural difference may have inadvertently increased aversiveness, potentially contributing to longer S– observing times. These findings highlight the complex interplay between contingency types, schedule arrangements, and observing behavior in human participants.

Our experiment incorporated punishment contingencies based on Pergher's (2007, Experiment 1) findings. In Pergher's study, participants under reinforcement vs. extinction schedules continuously pressed the space bar, preventing the execution of programmed reversals due to unmet learning criteria. To address this, we replaced the extinction component with punishment to generate a discriminated refrain‐from‐responding pattern. This approach proved effective, as evidenced by comparing P4's results with those of the other participants. Those who were exposed to reinforcement vs. punishment schedules met the learning criteria in fewer sessions during initial training and the three programmed reversals. Notably, this appears to be the first experiment evaluating functional class formation using punishment contingencies.

Our use of mild aversive consequences provides a viable alternative for situations where reducing response frequency is challenging. These results suggest that carefully implemented punishment contingencies can effectively shape behavior and facilitate learning in discrimination tasks, potentially expanding research avenues. However, punitive contingencies may limit the comparability, generalizability, and interpretation of our findings. Future studies should systematically compare reinforcement and extinction schedules when examining eye movements during simple discrimination training with contingency reversals. This approach would offer a more comprehensive understanding of observing behavior across different contingency types and enhance the generalizability of results.

Our study focused mainly on comparing S+ and S– observing responses. Huziwara et al. (2015) conducted an experiment in which six college students were taught successive simple discriminations. Using two sets of four stimuli each as S+ and S–, they found longer observing durations for S– than for S+. However, these results may have been an experimental artifact of their go/no‐go procedure. Stimuli were presented for up to 5 s, with points awarded for responding within this time limit for S+ components and for withholding responses in S– components. This aspect likely encouraged participants to respond quickly to S+ (go) and wait for S– (no‐go), artificially inflating S– exposure time. Such differences in stimulus exposure during the task can bias observing duration results, potentially obscuring the influence of discriminative functions (Huziwara et al., 2015). The present experiment addressed this issue by employing a free‐operant procedure instead of a forced‐choice procedure, allowing better control over stimulus exposure time.

Free‐operant procedures are well suited for studying eye movements in simple discrimination tasks. However, most experiments in this area focus on teaching conditional discrimination (e.g., Braaten & Arntzen, 2022; Hansen & Arntzen, 2021; Huziwara et al., 2016; Sadeghi & Arntzen, 2018), often using forced‐choice procedures such as MTS. These studies have advanced our understanding of how stimuli acquire discriminative control (e.g., Braaten & Arntzen, 2022; Dube et al., 2006, 2010; Huziwara et al., 2016). However, some findings appear inconsistent. For instance, Dube et al. (2006) reported that participants who observed all components of the sample stimulus for longer durations achieved higher accuracy in a MTS task teaching conditional relations based on physical similarity. In contrast, Hansen and Arntzen (2021) found that longer S+ observing time than S– in MTS trials did not reliably predict success in equivalence class formation tests. Further investigation is needed into how forced‐choice procedures influence observing patterns in MTS tasks. More controlled studies of observing responses could clarify certain performances in conditional discrimination tasks, such as overselectivity, which has been linked to errors in MTS tasks (Dube & McIlvane, 1999).

An intriguing finding in the present experiment relates to the practice effect (Schroeder, 1970), which typically involves a decrease in stimulus observing time as training progresses, suggesting that increased proficiency correlates with reduced observing time. This effect has been consistently reported in both simultaneous simple discrimination training (e.g., Pessôa et al., 2009; Schroeder, 1970) and conditional discrimination studies (e.g., Braaten & Arntzen, 2022; Huziwara et al., 2016). However, our results diverged from this trend because none of our participants exhibited a consistent decrease in stimulus observing time. Although the absence of a consistent practice effect in our data contrasts with previous findings (e.g., Schroeder, 1970), this could indicate that the type of task or contingency employed in our experiment alters the typical learning trajectory. Future studies should explore how these variations affect the development of discriminative control and whether practice effects emerge under different experimental conditions.

The relation between observing responses and manual choice responses has been documented in previous studies (e.g., Pessôa et al., 2009; Schroeder, 1970). Pessôa et al. examined participants' eye movements during simultaneous simple discrimination tasks involving four stimuli: two 2‐dimensional stimuli (square and circle) and two 3‐dimensional (cube and cylinder), 2D and 3D figures, respectively. Participants selected stimuli presented in the four corners of a monitor, with one 2D and one 3D stimulus functioning as S+ in each trial. The results showed a preference for choosing the 3D S+ over the 2D S+, with longer observing durations for the chosen S+ than for the other stimuli. Based on these findings, one could predict that participants would observe S– longer than S+ and respond more frequently to S– components (i.e., contrary to the new contingencies) in sessions immediately following a reversal. Such a pattern of manual choices in relation to observing responses could help explain the typical error patterns often described in simple discrimination training tasks with contingency reversals. However, our findings did not support this assumption. Considering P4's data in Figure 3, there was no evidence that S– was observed longer than S+ in any of the three reversals. In contrast, P5 exhibited longer S– observation in the second and third reversal sessions (see triangles above the circles in Figure 4). In addition, this pattern of observing responses was not accompanied by a high frequency of responses to S– components. In the experiment, manual and observing responses were independent: Participants could press the bar without necessarily looking at S+. Future studies could further investigate how observing responses are distributed across different task elements beyond the discriminative stimuli. For instance, an S+ presentation may reinforce observing responses that are directed toward the point counter rather than the discriminative stimulus itself.

The divergent patterns of space bar presses observed in P4 and P5 likely stem from their exposure to different schedules: reinforcement/extinction for P4 and reinforcement/punishment for P5. Whereas human studies typically use reinforcement vs. extinction schedules to establish reversals (e.g., Canovas et al., 2019; Lionello‐DeNolf et al., 2008; Postalli et al., 2015), P4's results suggest that this approach requires more sessions to achieve learning criteria than the reinforcement/punishment schedules. For context, Tomanari et al. (2007) reported an average of 40 sessions to reach the learning criterion (DI of 0.75 in one session) using reinforcement/extinction schedules. In contrast, our Study 1's initial training phase reached the criterion (DI of 0.70 in three consecutive sessions) in an average of four sessions. Interestingly, P5 exhibited longer S– observing durations than P4, possibly because observing S– may have prevented manual responses that could have led to point loss.

In summary, we analyzed the formation of functional classes through simple discrimination reversals, a process initially observed in pigeons and replicated with human participants in this study, alongside the monitoring of eye movements to assess whether observing responses align with the development of these functional classes. The results highlight the influence of discriminative stimuli on observing patterns, where changes in the duration of observing responses correlated with the establishment or reversals of discriminative functions for most participants. Additionally, most participants frequently observed S– for longer than S+. However, the differences in observing patterns between our study and previous research, such as Tomanari et al. (2007), point to the influence of contingency reversals on observing behavior. Our use of reinforcement and punishment schedules may have heightened the aversiveness of the S–, contributing to the longer observing durations seen in some of our participants.

In contrast to previous reinforcement/extinction schedules, using aversive contingencies in our study proved effective in facilitating learning, especially in participants like P5, who required fewer sessions to meet the learning criteria. This finding suggests that including mild aversive contingencies in discrimination tasks may provide a viable alternative when reducing response frequency is challenging, expanding potential applications of these procedures.

Additionally, our results suggest that free‐operant procedures may offer better control over stimulus exposure and observing duration than forced‐choice methods like MTS. This has important implications for future studies examining the influence of contingencies and task structures on observing behavior. Further investigation is needed into the factors that drive changes in observing responses, including whether contingency reversals and aversiveness levels systematically alter observing durations and manual choice responses.

In conclusion, this experiment contributes to our understanding of functional class formation and observing behavior in humans. We have highlighted the complex dynamics at play during discrimination learning tasks by examining the interaction between discriminative stimuli, contingency types, and observing responses. Future research should focus on refining experimental designs to address unanswered questions about the mechanisms behind these processes, particularly regarding the influence of contingency reversals and the long‐term effects of practice on observing behavior.

## AUTHOR CONTRIBUTIONS

N.K.P. and G.T. jointly conceived the study and designed the experiment. N.K.P conducted the experiments. N.K.P., E.M.H, and G.Y.T collaborated on data analysis and interpretation of results. E.M.H. wrote the initial draft of the manuscript, which N.K.P and G.T. critically revised. The authors approved the final version of the manuscript. The three authors are equally accountable for all aspects of the work and its integrity.

## CONFLICT OF INTEREST STATEMENT

The authors declare they have no conflict of interest.

## ETHICS APPROVAL

Proper informed consent was obtained, and the appropriate ethics review boards approved the study design.

## Data Availability

Raw data and supplementary materials are available from the authors upon request.
